# Oxidative stress and antioxidant status in patients with complicated urolithiasis

**Published:** 2016

**Authors:** E Ceban, P Banov, A Galescu, V Botnari

**Affiliations:** * Department of Urology and Surgical Nephrology, „Nicolae Testimiţanu” State University of Medicine and Pharmacy, Chisinau, Republic of Moldova; ** Clinic of Urology, Republican Clinical Hospital, Chisinau, Republic of Moldova

**Keywords:** oxidative stress, antioxidant status, complicated urolithiasis, kidney stone disease

## Abstract

In recent years, intense efforts have been made to clarify the pathogenesis of urolithiasis, which affects more than 10% of the population of developed countries. Currently, a number of studies have assumed a key role in the pathogenesis of oxalate urolithiasis, which is the most common one that belongs to the active forms of oxygen generated in the kidney, as a result of the activation of free radical oxidation that occurs in the interaction of calcium oxalate crystals with renal tubular epithelial cells. In the current work, oxidant and antioxidant status were assessed in the blood of patients with complicated urolithiasis pre - and post surgery. The surgical treatment of complicated urolithiasis leads a decrease of the oxidative stress and an increase in the potential of antiradical and antiperoxidative protection.

## Introduction

In recent years, intensive efforts to clarify the etiopathogenesis of renal lithiasis (RL) have been applied, a disease which affects more than 10% of the population in developed countries causing high disability [**1**,**2**]. In spite of the significant progress in the treatment of RL, due to the widespread use of modern methods applied in solving the renal ureteral calculi (ESWL, PCNL, high and low endourology methods combined with contact lithotripsy, inclusive and YAG laser, laparoscopic and surgical methods), which allow the release of calculi successfully, the pathophysiological mechanisms that contribute to the development of nephrolithiasis, have not been definitively elucidated, a fact that leads to new calculi appearance, i.e. recurrence of disease [**2**].

According to the specialty contemporary literature, in a high number of studies it was currently assumed that the key role in the pathogenesis of oxalate lithiasis, which is the most common, belongs to the active forms of oxygen, generated in the kidneys, due to the activation of oxidative free radicals, that take place due to crystals of calcium oxalate interaction with renal tubular epithelial cells. Direct and indirect evidence for this suggestion have been demonstrated both in vitro, in cell cultures that mime various nephron units, as well as in vivo in animal experiments and clinical observations [**3**-**7**].

Chronic calculus pyelonephritis has a particularly important role in RL recurrence, which still occurs in its pathogenesis cellular oxidation processes activation with the formation of free radicals [**13**-**16**]. Many authors have demonstrated that immunosuppression of immune status has an important role in the pathogenesis of CPN and that immunosuppression is involved in the chronic process [**1**,**2**,**8**,**13**-**16**] and recurrence of urolithiasis.

The aim of the current study was to assess the oxidant and antioxidant status of blood in patients with complicated urolithiasis pre - and postoperatively.

## Material and methods

The study was conducted on a group of patients with complex renal lithiasis treated in “N. Testimiţanu” Urology and Surgical Nephrology Department of the Republican Clinical Hospital during the years 2010 and 2014.

Antioxidant system parameters and indicators of oxidative stress were assessed in 58 patients with various forms of urolithiasis surgically treated and in 30 people in the control group. Both groups were homogenous according to gender and age. People in the control group were actually healthy. Thus, the organization of the study allowed the highlighting of specific pathological changes for complicated urolithiasis that required surgery, compared with healthy people.

Examinations conducted in patients suffering from renal lithiasis provided important information concerning the pathological changes in the antioxidant system.

The intensity of oxidative stress and antioxidant system changes were examined by dosing the following specific biochemical indices:

The level of malonic dialdehyde (MDA) was determined according to the procedure described by Atasayar S. and coauthors [**8**]. The method is based on a spectrophotometric determination of the trimetinic colored complex formed from the MDA interaction with thiobarbituric acid. MDA content (micromol/ l) is calculated based on the molar absorption coefficient = 1.56 Σ 105 mol • cm-1.

Determination of advanced oxidation protein products (AOPP) was performed according to the procedure described by Hong Yan Li et al. [**9**]. The method is based on the specific interaction of AOPP with potassium iodide in an acidic medium. The calculation is done according to the calibration curve built on successive dilutions of sol. standard of chloramine-T (0-100 μmol/ l / L) and is expressed in μmol/ l / l chloramine-T equivalents.

The dosage of nitric oxide in the biological material was carried out according to the procedure described by Метельская В. А. and Гуманова Н. Г. [**10**]. The method is based on the reduction of nitrate to nitrite, which interacts with the Griss reagent. The content of the final product is determined spectrophotometrically, and the calculation is carried out according to the calibration curve, built on successive dilutions of the stock standard solution of sodium nitrite (10 mmol).

Determination of glutathione reductase (EC 1.6.4.2) in the biological material was carried out according to the method described by Власова С.Н., Шабунина Е.И. and Переслегина И.А. in the modification V. Gudumac and coauthors [**11**]. This method is based on determining the speed increase in the level of reduced glutathione formed in the enzymatic reaction, which is assessed by the specific reaction with 5,5’-dithiobis-2-nitrobenzoate. GR activity is expressed in µmol DTNB/ min•l.

The dosage of glutathione peroxidase activity (EC 1.11.1.9) in the biological material was performed according to the method described by Wendel A. in the modification Gudumac V. and coauthors [**11**]. The method is based on determining the speed decrease of the level of reduced glutathione (GSH) in the reaction medium with the use 5,5’-dithiobis-2-nitrobenzoate. The reaction product is estimated spectrophotometrically, and the GPO activity is expressed in nmol of reduced glutathione per second in 1 serum - nmol/ s.l.

The activity of glutathione-S-transferase (EC 2.5.1.18) in the biological material was estimated in accordance with the procedure described by W. H. Habig and coauthors in the modification V. Gudumac and coauthors [**11**]. The method is based on the ability of glutathione-S-transferase to catalyze the condensation reaction of reduced glutathione with 1-Cl-2,4-dinitroclorbenzol (1-Cl-2,4-DNCB). The reaction product of 1-S-glutathionyl-2,4-dinitroclorine-benzol is determined at 340 nm spectrophotometric rider, and enzyme activity is expressed in nmol/ s·l

The activity of superoxide dismutase (SOD) (EC 1.15.1.1) was estimated according to the procedures described by Дубинина Е. Е. and Матюшин Б. Н. in the modification V. Gudumac and coauthors [**11**]. The method is based on the inhibition of the reduction of nitroblue tetrazolium salt in the system comprising the phenasinmetasulphate and NADH by SOD. Reducing NBT blue colored formazan is formed. The color intensity is directly proportional to the enzyme activity which relates to ml serum.

Catalase activity (EC 1.11.1.6) was assessed according to the process described by Королюк М. А. and coauthors [**12**] in the modification V. Gudumac and coauthors [**11**]. Determination of catalase activity is based on the ability of the enzyme to cleave the hydrogen peroxide into water and molecular oxygen. The hydrogen peroxide interacts with the ammonium molybdate to form a yellow compound. The color of the solution decreases with the decomposition of the hydrogen peroxide. The degree of fading in a certain time period correlates with the activity of the enzyme that is expressed in µmol/ s·l of serum. 

Assessing the level of ceruloplasmin (CP) was performed according to Колб В. and coauthors in modification V. Gudumac and coauthors [**11**]. The method is based on the property of CP to oxidize various compounds, including p- phenylenediamine. Its oxidation products are formed of the blue violet color, which is measured spectrophotometrically. The color intensity is proportional to the content of CP, which is expressed in mg/ l.

The content of thiol groups of proteins in the biological material was determined according to the method described by G. L. Ellman and Hu M. L. in modification V. Gudumac and coauthors [**11**]. The method principle is based on the interaction between Ellman’s reagent (5,5’-dithiobis- (2-nitrobenzoic acid) and thiol groups of proteins forming a colored compound, and the results’ reading is carried out by using the spectrophotometric rider at 412 nm.

Total protein determination (according to Lowry). It is performed according to the process described by V. Gudumac and coauthors [**11**]. The principle of the method is based on the ability of copper compounds of proteins to reduce the Folin reagent with the formation of colored products of the reaction. The color intensity is directly proportional with the amount of protein in the material under investigation.

SPSS program (version 20.0) was used for the statistical processing of the data. The descriptive and comparative statistics was also used. Data were presented according to the formula Mean ± Standard formula Error of Mean. Kolmogorov-Smirnov and Mann-Whitney nonparametric tests were used for the comparative analysis between the study group and the control group. Sign and Wilcoxon tests were used for the pre- and postoperative assessment of changes in the studied group. A significant threshold for comparison was set in 5% (p <0.05).

## Results and discussions

Biochemical investigations carried out in patients suffering from renal lithiasis provided important information regarding the changes in the intensity of free radicals oxidation and antioxidant system activity (**Table 1**). Comparative analysis of the values of indicators of oxidative stress and antioxidant system activity in patients with urolithiasis and in the control group, carried out under Kolmogorov-Smirnov and Mann-Whitney nonparametric tests, showed the presence of authentic statistical differences between both studied groups.

**Table 1:** Free radicals oxidation intensity and antioxidant system activity changes in renal lithiasis

Index	Group I	Group II	p1	Group III	p2
NO (μmol/ l)	80,94 ± 1,39	81,42 ± 2,09	+0,6%, p>0,05	104,17 ± 2,51	+28% p<0,001
DAM (μmol/ l)	13,31 ± 0,68	20,57 ± 1,67	+70%, p<0,001	14,65 ± 0,69	-29%, p<0,001
AGE (μg/ ml)	550,53 ± 26,57	567,6 ± 33,89	+3%, p>0,05	409,10 ± 24,89	-28%, p<0,001
Ceruloplasmin (μmol/ l)	353,57 ± 11,29	408,3 ± 14,19	+14%, p<0,01	371,91 ± 14,24	-9%, p<0,05
SOD (μmol/ s.l)	863,88 ± 19,72	809,7 ± 42,22	-7%, p<0,05	851,87 ± 20,09	+5%, p ≈ 0,53
Catalysis (μmol/ s.l)	11,39 ± 1,00	10,77 ± 1,31	-5%, p>0,05	10,63 ± 0,76	-1%, p≈0,48
GR (nmol/ s.l)	475,77 ± 26,81	511,6 ± 65,61	+8%, p>0,05	402,46 ± 22,41	-21%, p≈0,74
GPO (nmol/ s.l)	549,98 ± 49,95	443,4 ± 58,04	-20%, p<0,05	666,3 ± 114,39	+50%, p<0,01
G-S-T (nmol/ s.l)	119,09 ± 9,09	106,1 ± 14,14	-13%, p<0,05	81,48 ± 10,17	-23%, p≈0,35
Protein-SH groups (μmol/g prot.)	7,58 ± 0,34	7,29 ± 0,62	-6%, p<0,01	3,40 ± 0,21	-53% p<0,001
S-nitrosothiol (μmol/ l)	3,84 ± 0,09	3,74 ± 0,12	-3%, p>0,05	3,56 ± 0,13	-5%, p≈0,22
*NOTE: a) Group I – control, Group II – renal lithiasis, Group III – renal lithiasis + surgical treatment;*					
*b) p1 - the veracity of statistical differences between group I vs. II according to Kolmogorov-Smirnov test;*					
*c) p2 - the veracity of statistical differences between group II vs. III according to the Wilcoxon test.*					

The research results revealed an increased intensity of oxidative stress in patients with renal lithiasis compared to healthy individuals, demonstrated by an important accumulation of the end product of lipid peroxidation – of the malonic dialdehyde (+70%, p <0.001), and the lowering of the Protein-SH groups (+6%, p<0,01). In compensation, the ceruloplasmin non-enzymatic antioxidant level increased by 14% (p <0.01) in order to maintain normal redox potential in conditions of the studied disease.

This increase cannot balance the enzyme compartment depletion of SAO, identified in patients with urolithiasis. A conclusive statistical decrease of SOD activity (-7%, p <0.05) and of non-veridical catalase was established (-5%, p> 0.05), enzymes which ensured a primary elimination of superoxide radical and of hydrogen peroxide and limited, in this way, the production of the most harmful radical - hydroxyl.

A significant reduction of the enzyme activity involved in the neutralization of organic peroxides - glutathione peroxidase (-20%, p <0.05) and glutathione-S-transferase (-13%, p <0.05) was also found, which resulted in the eliminating capacity reduction of the membrane lipid peroxidation products and biological liquids and their accumulation.

Thus, we can conclude that, pathogenetic links oxidative stress by lipid peroxidation, particularly, of polyunsaturated fatty acids, which can result in significant damage to biological membranes and organelles destruction and/ or cells, can certainly be included in the urolithiasis. The process is amplified due to proteins oxidative damage which has severe repercussions on organ function, evolution and the probability of developing complications, including the fatal ones of acute or chronic renal insufficiency. The activity decrease of antioxidant enzymes (SOD, GPO and G-S-T) deepens the aforementioned disorders, by the organism’s inability to counter the damaging effects of oxidative stress.

The assessment of dynamics of oxidative stress intensity and of antioxidant system activity in patients suffering from renal lithiasis showed an improvement of these parameters after surgical treatment (**Table 1**). Sign and Wilcoxon tests allowed the assessment of changes determined by the surgical treatment of complicated renal lithiasis. Since the results of both tests were substantially similar concerning the degree of statistics truthfulness, the paper presented authentic statistical differences according to the Wilcoxon test.

The surgical removal of renal calculus diminished the oxidative stress’ strength. Oxidation intensity decreased with free radicals determining the reduction processes of lipid peroxide oxidation, confirmed by the statistically significant decrease of malonic dialdehyde concentration after surgery (29%, p <0,001) and reaching the values close to those specific to the control group (**Table 1**).

The improvements mentioned above might be determined by several factors. One of them could be the non-enzymatic antioxidants content normalization after the surgical treatment for renal calculus removal. Our researches identified a significant decrease (9%, p <0.05) in the level of ceruloplasmin in the operated patients, which practically reached the values identified in healthy individuals.

Another important factor was the restoration of antioxidant enzymes functionality. The biochemical data established that SOD activity, a first line enzyme of antiradical protection, increased up to 851.87 ± 20.09 μmol/ s.l, a value close to the reference one detected in healthy individuals (851.87 ± 20.09 μmol/ s.l). Catalysis activity was not affected by the surgery, as well as by the malady, and maintained at values close to those found in the control group (10.63 ± 0.76 μmol/ s.l).

The surgical treatment of urolithiasis determined a particularly important increase of glutathione peroxidase activity (by 50%, p <0.01), whereas the values of glutathione-S-transferase and glutathione reductase remained quasi constant during the period of monitoring (p> 0, 05). Also, in surgically treated patients, the number of sulfhydryl groups of serum proteins (53%, p <0.001) was significantly decreased.

## Conclusions

Thus, the surgical removal of the renal calculus may eliminate some triggering factors of oxidative stress, which improve laboratory indices of free radical oxidation in patients treated this way. Simultaneously, there was a recovery in the level and activity of non-enzymatic components and of antioxidant enzyme system with the increase of anti-radical and anti-peroxide potential protection of the kidneys and of the body in general. These changes created a solid molecular foundation for organ functionality restoration.
